# Resistin, a fat-derived secretory factor, promotes metastasis of MDA-MB-231 human breast cancer cells through ERM activation

**DOI:** 10.1038/srep18923

**Published:** 2016-01-05

**Authors:** Jung Ok Lee, Nami Kim, Hye Jeong Lee, Yong Woo Lee, Su Jin Kim, Sun Hwa Park, Hyeon Soo Kim

**Affiliations:** 1 Department of Anatomy, Korea University College of Medicine, Seoul, Korea

## Abstract

Resistin, an adipocyte-secreted factor, is known to be elevated in breast cancer patients. However, the molecular mechanism by which resistin acts is not fully understood. The aim of this study was to investigate whether resistin could stimulate invasion and migration of breast cancer cells. Here, we report that resistin stimulated invasion and migration of breast cancer cells as well as phosphorylation of c-Src. Inhibition of c-Src blocked resistin-induced breast cancer cell invasion. Resistin increased intracellular calcium concentration, and chelation of intracellular calcium blocked resistin-mediated activation of Src. Resistin also induced phosphorylation of protein phosphatase 2A (PP2A). Inhibition of c-Src blocked resistin-mediated PP2A phosphorylation. In addition, resistin increased phosphorylation of PKCα. Inhibition of PP2A enhanced resistin-induced PKCα phosphorylation, demonstrating that PP2A activity is critical for PKCα phosphorylation. Resistin also increased phosphorylation of ezrin, radixin, and moesin (ERM). Additionally, ezrin interacted with PKCα, and resistin promoted co-localization of ezrin and PKCα. Either inhibition of c-Src and PKCα or knock-down of ezrin blocked resistin-induced breast cancer cells invasion. Moreover, resistin increased expression of vimentin, a key molecule for cancer cell invasion. Knock-down of ezrin abrogated resistin-induced vimentin expression. These results suggest that resistin play as a critical regulator of breast cancer metastasis.

Obesity is associated with an increased risk of breast cancer, although its exact mechanism has not been determined^1^,^2^,^3^. Breast cancer is the most common malignancy among women^4^. Therefore, it is critical to investigate possible prognostic factors as well as therapeutic targets for breast cancer. Increased estrogen level has been suggested to be a possible risk factor for breast cancer^5^. However, increased estrogen level alone cannot account for the association between obesity and the development of breast cancer^6^. Adipocytes can secrete metabolites, hormones, and cytokines, collectively known as adipocytokines. Examples of these include leptin, adiponectin, complement components, tumor necrosis factor-α, interleukin-6, and resistin^7^,^8^,^9^. Changes in adipokine levels caused by obesity are associated with the development and progression of several malignancies such as breast, gastric, colorectal, and esophageal cancers^9^,^10^,^11^.

Resistin has been shown to be involved in inflammatory processes such as atherosclerosis as well as various cancers such as colorectal, prostatic, and endometrial cancers^12^,^13^,^14^,^15^,^16^. In a previous study, serum resistin level was found to be significantly higher in breast cancer patients as compared with that in normal subjects^17^. Furthermore, it has been reported that high resistin expression in breast cancer tissue is associated with malignancies, postmenopausal breast cancer, and poor cancer prognosis^18^,^19^,^20^. Although accumulating evidence suggests that resistin plays an important role in the progression of cancers, the molecular mechanisms by which it acts has not been fully evaluated.

Ezrin is a member of the ezrin, radixin, and moesin (ERM) protein family which links F-actin to cell membrane proteins following phosphorylation^21^,^22^,^23^,^24^. This linker function suggests that ezrin is essential for many fundamental cellular processes including determination of cell shape, polarity, surface structure, cell adhesion, motility, cytokinesis, phagocytosis, and signaling pathways involved in membrane transport^25^,^26^,^27^,^28^. Recent studies have revealed that ezrin may also have an important role in tumorigenesis, cancer cell invasion, cross-cell signaling, and tumor metastasis, possibly via regulation of adhesion molecules^29^,^30^,^31^,^32^,^33^. Although ezrin is indispensable for tumor cell metastasis in osteosarcomas^34^, breast cancer^35^, and prostatic cancer^36^, the detailed molecular mechanisms regarding the involvement of ERMs in cancer progression remain unclear.

In this study, we found that resistin increased ERM signaling network. These findings provide novel insights into how resistin contributes to the metastatic behavior of breast cancer cells.

## Results

### Resistin increases invasion and migration of breast cancer cells

**Figure 1 f1:**
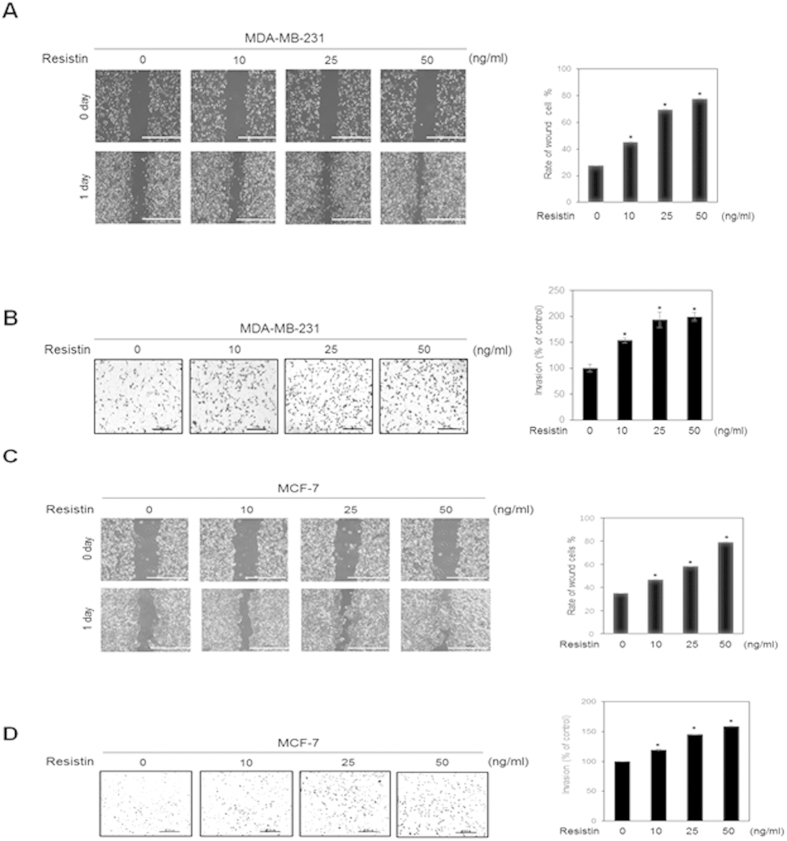
Resistin increases invasiveness of human breast cancer cells. (**A**) MDA-MB-231 cells were seeded on a 6-well plate, and incubated until confluent monolayers were formed. A cell-free space was created by scraping through the monolayer. Migration was induced by treatment with 0, 10, 25, or 50 ng/ml resistin for 24 h. (**B**) MDA-MB-231 cells were stained, and randomly chosen fields were photographed at 40× magnification. The number of cells invading the lower surface of the monolayer was counted. Data are presented as mean ± SD from 3 independent experiments. (**C**) MCF-7 cells were seeded on a 6-well plate and incubated until a confluent monolayer was formed. A cell-free space was created by scraping through the monolayer. Migration was induced by treatment with 0, 10, 25, or 50 ng/ml resistin for 24 h. (**D**) MCF-7 cells were stained, and randomly chosen fields were photographed at 100× . The number of cells invading the lower surface was counted. The mean ± S.D. of 3 independent experiments is presented. *P < 0.05 vs. untreated control.

To determine whether resistin affects the migration and invasion of MDA-MB-231 human breast cancer cells, wound-healing and invasion assays were performed. As demonstrated by the scratch wound assays, MDA-MB-231 cell migration was significantly increased following resistin treatment (Fig. 1A). In addition, resistin also increased MDA-MB-231cells invasion at a concentration of 10 ng/ml. The degree of invasion was further increased at higher concentrations of 25 ng/ml and 50 ng/ml (Fig. 1B). Resistin had no significant cytotoxic effect on cell viability up to 50 ng/ml in MTT assays (Data not shown). Tumor cells are known to have heterogeneity. To confirm the metastatic effect of resistin on the breast cancer cells, we tested another breast cancer MCF-7 cells (Supplementary Fig. 1 A,B,C and D). Resistin increased both migration and invasion of MCF-7 cells in a dose-dependent manner (Fig. 1C[Fig f1]D). These results demonstrated that resistin promotes invasion and migration of breast cancer cells.

### Intracellular calcium is required for resistin-induced phosphorylation of c-Src

**Figure 2:**
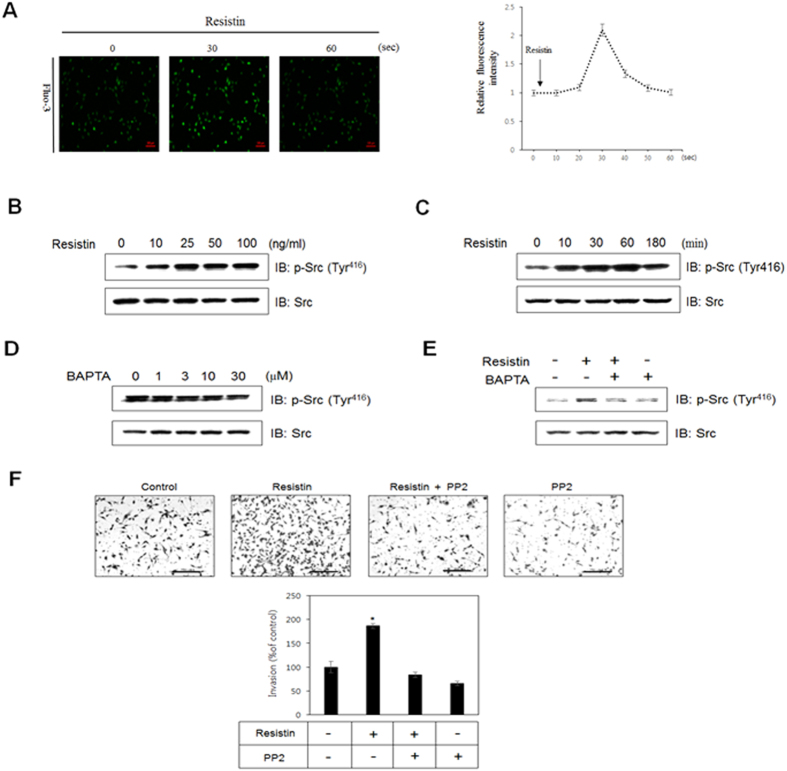
Intracellular calcium is involved in resistin-induced phosphorylation of c-Src. (**A**) MDA-MB-231 cells were pre-incubated in Fluo-3 AM (5 μM) for 45 min. Calcium response was measured by confocal microscopy, where calcium concentration correlated with fluorescent intensity. (**B**) Cells were stimulated for 60 min at several concentrations of resistin. Western blots were performed on cell lysates (30 μg), and the membrane was probed for phospho-c-Src with c-Src as the control. (**C**) Cells were incubated with 25 ng/ml resistin for the indicated times. Western blots were conducted on cell lysates, and the membrane was probed for phospho-c-Src with c-Src as the control. (**D**) After being serum-starved overnight, cells were treated with the indicated concentrations of resistin for 30 min. Western blots were conducted on cell lysates, and the membrane was probed for phospho-c-Src with c-Src as the control. (**E**) Cells were incubated for 30 min with 25 ng/ml resistin in the presence of BAPTA-AM. Western blots were conducted on cell lysates, and the membrane was probed for phospho-c-Src with c-Src as the control (**F**) Each column represents the average of 4 replicates of the Matrigel invasion assays. The results are displayed as mean ± SD from 3 independent experiments. *P < 0.05 vs. untreated condition.

Calcium is a crucial regulator of cell migration and invasion^37^. To investigate the effects of resistin on intracellular calcium, calcium concentrations were measured in the presence of resistin. Results indicated that intracellular calcium concentration was increased 30 seconds after addition of resistin (Fig. 2A). c-Src is a critical link between multiple signaling pathways that regulate proliferation, invasion, survival, metastasis, and angiogenesis^38^,^39^,^40^. To evaluate the possible involvement of c-Src during resistin treatment, we measured c-Src phosphorylation. Phosphorylation of c-Src increased in a dose- and time-dependent manner in the presence of resistin (Fig. 2B,C). It has been reported that calcium regulates the activity of c-Src^41^. We therefore assessed the effect of BAPTA-AM, a calcium-selective chelator, on c-Src phosphorylation. We found that intracellular calcium chelation decreased phosphorylation of c-Src in a dose-dependent manner (Fig. 2D). We next investigated the role of calcium in resistin-mediated phosphorylation of c-Src. Chelating of intracellular calcium inhibited resistin-mediated phosphorylation of c-Src, suggesting that calcium is required for c-Src phosphorylation (Fig. 2E). To assess the effect of c-Src on resistin-induced invasion, we pre-treated breast cancer cells with the c-Src inhibitor PP2, and analyzed the invasion ability of MDA-MB-231 cells via a transwell assay. Pre-treatment with PP2 decreased resistin-induced MDA-MB-231 cells invasion (Fig. 2F), suggesting that this process is mediated by c-Src. Our data indicated that resistin stimulates invasion and migration of MDA-MB-231 cells through calcium-dependent c-Src pathway.

### PP2A is involved in resistin-induced breast cancer cells invasion

**Figure 3:**
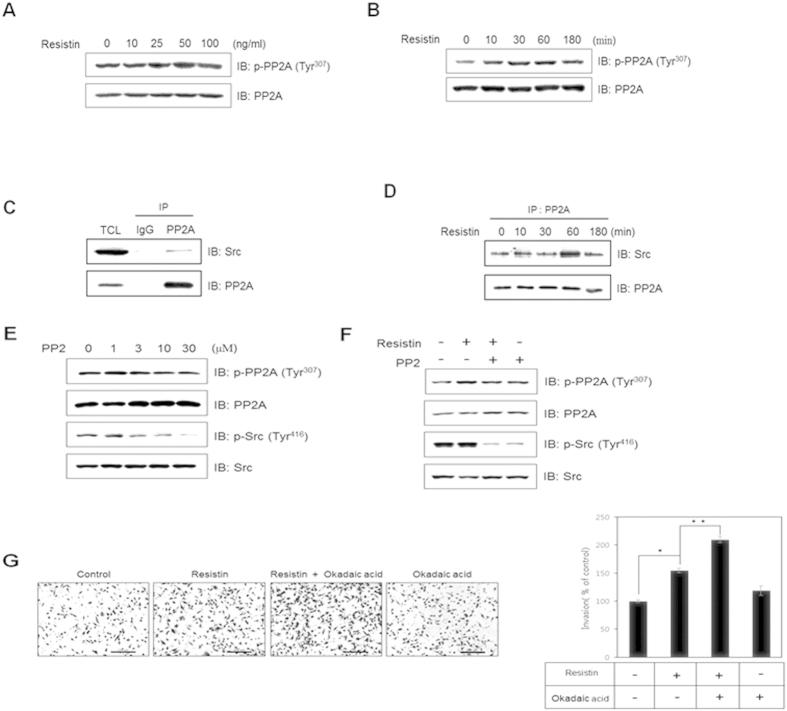
PP2A is involved in resistin-induced breast cancer cells invasion. MDA-MB-231 cells were (**A**) treated with different doses of resistin and cultured for 1 h, or (**B**) treated with resistin (25 ng/ml) for the indicated time points. Western blots were conducted on cell lysates (30 μg), and the membrane was probed for phospho-PP2A with PP2A serving as the control. (**C**) MDA-MB-231 cells were immunoprecipitated with anti-PP2A antibody, followed by western blots against c-Src and PP2A. (**D**) Cells were treated with 25 ng/ml resistin for the indicated times, and cell lysates were immunoprecipitated with anti- PP2A antibody. Western blots were performed to detect protein expression of PP2A and Src. (**E**) Cells were treated with PP2 (0, 1, 3, 10, or 30 μM). Cell lysates (30 μg) were analyzed by western blots using antibodies against phospho-PP2A and phospho-c-Src, while PP2A and c-Src served as controls. (**F**) Cells were pre-treated with PP2 (10 μM) and incubated with 25 ng/ml resistin for 60 min. Cell lysates (30 μg) were analyzed by western blot using antibodies against phospho-PP2A and phospho-c-Src, while PP2A and c-Src served as controls. (**G**) For the Matrigel invasion assay, the number of cells invading through the Matrigel was represented by each column (4 replicates). The results are displayed as mean ± SD from 3 independent experiments. *P < 0.05 vs. untreated condition. **P < 0.05 vs. resistin-treated condition.

The catalytic subunit of PP2A is regulated by phosphorylation of Tyr 307, resulting in inactivation of the enzyme^42^,^43^. To assess the effect of resistin on PP2A activity, we measured phosphorylation of PP2A. We found that resistin increased PP2A phosphorylation in a dose- and time-dependent manner (Fig. 3A,B), suggesting that resistin reduces PP2A activity. It is known that c-Src directly phosphorylates PP2A^44^. Therefore, to elucidate the mechanism underlying resistin-mediated inhibition of PP2A activity, we next examined the interaction between c-Src and PP2A. We found that PP2A interacted with c-Src in MDA-MD-231 cells (Fig. 3C). Resistin increased this interaction in a time-dependent manner, suggesting that resistin facilitates interactions between c-Src and PP2A (Fig. 3D). To determine whether c-Src kinase regulates phosphorylation of PP2A, we measured the level of PP2A phosphorylation following treatment with the c-Src inhibitor PP2. Inhibition of c-Src decreased PP2A phosphorylation in a dose-dependent manner (Fig. 3E). In addition, inhibition of c-Src decreased resistin-mediated phosphorylation of PP2A, suggesting that c-Src is involved in resistin-induced inhibition of PP2A (Fig. 3F). To better understand the role of PP2A, we measured MDA-MD-231 cells cell invasion using the transwell assay. Results indicated that MDA-MD-231 cells pre-treated with the PP2A inhibitor okadaic acid exhibited enhanced resistin-induced invasion (Fig. 3G). These results suggest that resistin stimulates invasion of MDA-MB-231 cells by reducing PP2A activity.

### PKCα is involved in regulation of resistin-induced PP2A activity

**Figure 4:**
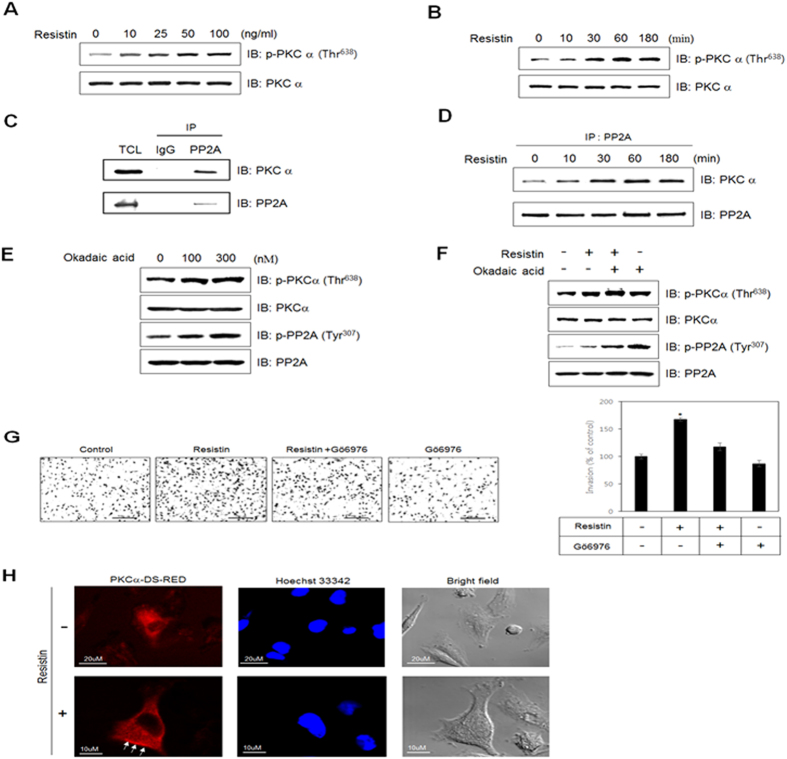
PKCα regulates resistin-induced PP2A activity. MDA-MB-231 cells were treated with (**A**) different doses of resistin and cultured for 1 h, or (**B**) treated with resistin (25 ng/ml) for the indicated times. Cell lysates (30 μg) were analyzed by western blots using antibody against phospho-PKCα, while PKCα served as the control. (**C)** Cells were immunoprecipitated with anti-PP2A antibody, followed by western blots with anti-PP2A and PKCα antibodies. (**D)** Cells were treated with 25 ng/ml resistin for the indicated times. The lysates were immunoprecipitated with PP2A antibody, followed by western blots with anti-PP2A and PKCα antibodies. **(E)** Cells were treated with okadaic acid. Cell lysates (30 μg) were analyzed by western blots using antibodies against phospho-PKCα and phospho-PP2A. PKCα and PP2A served as controls. (**F)** Cells were pre-treated with okadaic acid (100 nM), and then incubated with resistin for 60 min. Cell lysates were subjected to western blots, and probed for phospho-PKCα as well as phospho-PP2A; PKCα and PP2A served as controls. (**G**) The number of cells invading through the Matrigel are shown in columns, each representing the average of 4 replicates. (**H)** Representative images (PKCα-DS-RED, nuclei, and objective image) of PKCα-DS-RED-expressing cells treated with resistin for 30 min. Scale bars, 10 μm. The results are displayed as mean ± SD from 3 independent experiments. *P < 0.05 vs. untreated condition.

PKCα has been implicated in tumor growth and progression^45^. To evaluate the involvement of PKCα in PP2A activity, we first investigated whether resistin increases phosphorylation of PKCα. Indeed, resistin increased PKCα phosphorylation in a dose- and time-dependent manner (Fig. 4A,B). It was previously reported that PKCα is dephosphorylated by PP2A^46^. To investigate the relationship between PKCα and PP2A, we performed immunoprecipitation assays, and demonstrated that PP2A interacted with PKCα (Fig. 4C). Moreover, resistin was found to enhance this interaction in a time-dependent manner (Fig. 4D), suggesting that resistin regulates the interaction between PKCα and PP2A. To determine whether PP2A regulates phosphorylation of PKCα, we measured phosphorylation of PKCα following treatment with okadaic acid. Inhibition of PP2A activity increased the phosphorylation of PKCα, suggesting that PP2A enhances PKCα activity under resistin-treated conditions (Fig. 4E). We next assessed the effect of resistin on PKCα phosphorylation in the presence of okadaic acid. Inhibition of PP2A increased PKCα phosphorylation, suggesting that PP2A is involved in resistin-induced augmentation of PKCα activity (Fig. 4F). To investigate the role of PP2A on cancer metastasis, we measured invasion activity of MDA-MD-231 cells pretreated with okadaic acid, and found that inhibition of PP2A increased the resistin-induced breast cancer cells invasion (Fig. 4G). To confirm the effect of resistin on PKCα activation, we examined the subcellular localization of MDA-MD-231 cells expressing PKCα-DS-RED by immunocytochemistry. Treatment with resistin led to translocation of PKCα-DS-RED (arrow indicated parts) from the cytosol to the plasma membrane (Fig. 4H). These results suggest that resistin stimulates invasion of MDA-MB-231 cells by regulating the PP2A-mediated PKCα pathway.

### Resistin stimulates invasion of breast cancer cells through ERM

**Figure 5:**
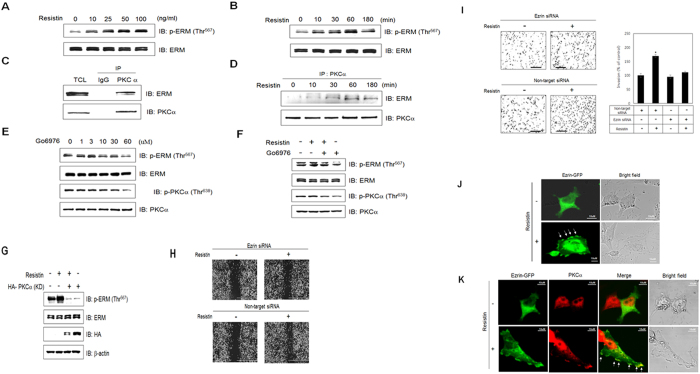
Resistin stimulates invasion of breast cancer cells through ERM. MDA-MB-231 cells were treated with (**A**) different doses of resistin and cultured for 1 h, or (**B**) treated with resistin (25 ng/ml) for the indicated times. Cell lysates (30 μg) were analyzed by western blots using antibody against phospho-ERM; ERM was used as the control. (**C**) Cells were immunoprecipitated with anti-PKCα antibody, followed by western blots with anti-ERM and PKCα antibodies. (**D**) Cells were treated with resistin for the indicated times, and the lysates were immunoprecipitated with anti-PKCα antibody, followed by western blots with anti-ERM and PKCα antibodies. (**E**) Cells were treated with Gö 6976. Cell lysates (30 μg) were analyzed by western blots using antibodies against phospho-ERM and phospho-PKCα; ERM and PKCα were used as controls. (**F**) Cells were pretreated with Gö 6976 (10 μM) and incubated with resistin for 60 min. Cell lysates were analyzed by western blots using antibodies against phospho-ERM and phospho-PKCα; ERM and PKCα were used as controls. (**G**) HA-tagged PKCα (KD)-expressing cells were treated with resistin for 1 h. Cell lysates were analyzed by western blots using anti-phospho-ERM and HA antibodies; ERM and β-actin were used as controls. (**H**) Cells were transfected with non-target or ezrin siRNA for 48 h. A cell-free space was created by scraping through the monolayer. Migration was induced by 25 ng/ml resistin for 16 h. (**I**) Effect of ezrin suppression on cell invasion. Cells were transfected with non-target or ezrin siRNA for 48 h. Cells were subjected to an invasion assay and stained, and randomly chosen fields were imaged at 100× magnification. The number of cells invading the lower surface was counted. (**J**) Representative images (ezrin and objective image) of ezrin-GFP-expressing cells treated with resistin for 1 h. Scale bars, 10 μm. (**K**) Representative images (ezrin-GFP, PKCα, and bright field) of cells treated with resistin for 1 h. Scale bars, 10 μm. *P < 0.05 vs. untreated condition.

ERM proteins provide a physical link from F-actin to membrane-associated proteins on the surface of cells. This linker function makes ERM essential for many fundamental cellular processes such as determination of cell shape, polarity, and cell adhesion. We first tested the effect of resistin on ERM activation. Resistin induced ERM phosphorylation in a dose-dependent manner in MDA-MB-231 cells (Fig. 5A). Resistin also increased ERM phosphorylation in a time-dependent manner (Fig. 5B). It was previously reported that ezrin is a downstream effector of PKCα that controls cell motility^47^. To confirm this, we investigated the interaction between PKCα and ERM, Results showed that ERM was able to interact with PKCα (Fig. 5C), and that resistin increased this interaction in a time-dependent manner (Fig. 5D). To confirm the involvement of PKCα in ERM activation, we measured ERM phosphorylation following treatment with Gö6976, a PKCα inhibitor. Inhibition of PKCα activity reduced ERM phosphorylation in a dose-dependent manner, suggesting that PKCα is involved in resistin-induced ERM activation (Fig. 5E,F). Furthermore, ERM phosphorylation failed to occur in PKCα kinase-defective (KD) mutants (Fig. 5G). Compare with cells transfected with non-target siRNA, the cells treated with ezrin siRNA showed a wider wound area after wound generation by resistin indicating a defect in migration (Fig. 5H). To define the contribution of ERM to the resistin-induced metastatic effect, an invasion assay was performed following ezrin knockdown. Ezrin siRNA-transfected cells significantly attenuated resistin-induced cell invasion (Fig. 5I). To further confirm the effect of resistin on ezrin activation, we examined subcellular localization of GFP-tagged ezrin following resistin treatment. Treatment with resistin led to translocation ezrin (indicated by arrow) from the cytosol to the plasma membrane (Fig. 5J). As shown in Fig. 5K, co-localization of ezrin (green signal) and PKCα (red signal) to the plasma membrane (indicate by arrow) was observed following resistin treatment. These results suggest that resistin promotes invasion and migration of MDA-MB-231 cells by regulating the PKCα-ezrin pathway.

### Ezrin is involved in resistin-induced vimentin expression

**Figure 6:**
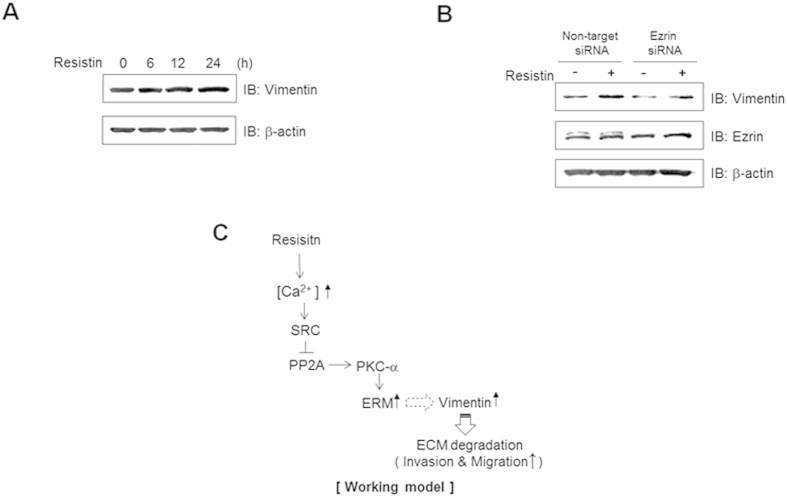
Ezrin is involved in resistin-induced vimentin expression . (**A**) MDA-MB-231 cells were treated with resistin (25 ng/ml) and cultured for the indicated times. Cell lysates (30 μg) were analyzed by western blots using antibody against vimentin; β-actin was used as a control. (**B)** Cells transfected with either non-target or ezrin siRNA were treated with resistin (25 ng/ml) for 24 h. Vimentin, ezrin, and β-actin were detected by western blots. (**C**) Schematic diagram of the mechanism of resistin.

Vimentin is a filamentous protein that controls cell shape changes during epithelial-mesenchymal transitions (EMT), and are strongly associated with cell invasion and poor tumor prognosis^48^. To gain insight into the roles of resistin on EMT, we evaluated the effect of resistin on vimentin expression. Administration of resistin induced an increase in vimentin expression (Fig. 6A). On the other hand, knockdown of ezrin by siRNA transfection significantly reduced resistin-induced expression of ERM proteins (Fig. 6B). Combined with all our data, we suggested the schematic diagram of the mechanism of resistin. Resistin triggers the activation of ERM pathway via calcium-mediated Src/PP2A/PKCα, which in turn induces the expression of vimentin (Fig. 6C). These results indicate that resistin triggers cell migration and invasion via activation of ERM proteins.

## Discussion

The objective of our study was to determine whether resistin directly regulates invasion of tumor cells, and if so, what are the molecular signaling pathways are involved in the process. Our principal finding was that ERM proteins promote resistin-mediated invasion of MDA-MB-231 cells. Association of ERM with enhanced tumor cell invasion raises several questions regarding the mechanism by which resistin can promote tumor cell invasion. The results of our study suggest that PKCα-mediated ERM activation plays a critical role in the progression of tumor cell invasion. Combined with a recent report showing that ERM is critical for tumor cell invasion, these findings implicate a role for ERM in resistin-mediated tumor cell invasion.

PKC has been implicated in a various types of tumorigenesis including cancer cell proliferation, migration, and invasion^49^. Among PKC isozymes, PKCα has been suggested to be important in breast cancers^50^,^51^. For example, PKCα expression is elevated in breast cancer tissues^41^. PKCα overexpression also increases the invasiveness of breast cancer cells^52^. However, the molecular mechanism underlying PKCα-mediated tumorigenesis is not known due to a lack of isozyme-specific tools. Our results showed that ERM induction was dependent on PKCα activity, suggesting that PKCα-controlled pathways represent an alternative mechanism by which ERM can stimulate cancer cell invasion. ERM proteins provide a physical link from F-actin to membrane-associated proteins, which are important for many cellular processes including cell motility, cytokinesis, and migration. Phosphorylation of ERM is important for its activation. Several protein kinase C kinases have been implicated in ERM activation^53^,^54^,^55^,^56^. In the present study, resistin increased phosphorylation of ERM through PKCα. Moreover, interactions between these two proteins were observed under resistin stimulation. Nevertheless, this study does not address the molecular target of resistin. This question can be answered by focusing on the relationship between PKCα and ERM, which we are currently investigating.

Some studies have proposed potential mechanisms connecting resistin with breast tumors. Accumulating evidence suggest that resistin exerts its neoplastic effect via two mechanisms. First, it acts directly on cancer cells by stimulating specific signaling pathways via an unknown receptor. Second, it may act indirectly on target cells by regulating inflammatory responses, influencing tumor angiogenesis, and modulating insulin sensitivity^57^. More mechanistic studies need to be conducted in order to understand this relationship at a molecular level. Adipocytes represent the majority of the breast tissue, and function as an active endocrine organ by secreting bioactive adipocytokines. In addition, adipocytes also regulates pathological processes such as carcinogenesis. In obesity-associated cancer, excess body weight has been proposed as a trigger for subclinical low-grade inflammatory state, where activation of pro-inflammatory adipocytokines may lead to carcinogenesis^58^,^59^. Importantly, resistin is found in inflammatory zones^59^, and elevated resistin levels are found in several malignancies such as breast and colorectal cancers. *In vitro* studies showed that resistin stimulates cancer cell proliferation through PI3K and MAPK signaling^60^,^61^. Another study demonstrated that resistin promotes the development of cancer through upregulation of proinflammatory cytokines^62^. Induction of matrix metalloproteinases (MMPs) may participate in tumor migration and invasion. In this study, we found that treatment of MDA-MB-231 cells with resistin led to a robust increase in the expression of vimentin, whereas induction of vimentin was not observed under ERM knockdown conditions. These results suggested that ERM may be involved in resistin-induced vimentin upregulation. Combined with the observation that vimentin is critical for tumor metastasis, the findings of our current study indicated that resistin induces tumor invasion via ERM-mediated vimentin induction. However, more studies are required to clarify the mechanistic role of resistin in the association between obesity and breast cancer. The concentration of resistin in humans ranges from 2 to 20 ng/ml^63^. In this experiment, we used 25 ng/ml of resistin, suggesting that our data may have physiological relevance.

Some studies have also investigated the metastatic effect of resistin *in vivo*. It was reported that resistin promotes chondrosarcoma metastasis and MMP2 expression through activation of AMPK/p38 signaling pathway and down-regulation of miR-519d expression in mouse models^64^. Mice that received anti-resistin antibodies showed decreased incidence of cancer development and metastasis^65^. It was proposed that resistin promotes cancer dissemination by maintenance and promotion of cell adhesion molecules^66^. Moreover, resistin influenced tumor progression via induction of pro-angiogenic proteins^67^. Together, these observations suggest that resistin is associated with cancer cell metastasis effect *in vivo*, but many questions remain to be answered.

Although we have provided clinical implications of resistin through the aforementioned experiments, there are still limitations in our study. Our experiments were all performed in an *in vitro* setting due to the mechanistic nature of the study. While our study results have provided molecular insights into breast cancer metastasis, our data are lacking in their physiological relevance. In addition, as we did not verify the disease phenotype in an ERM knock-down animal, we cannot conclude that metastasis of breast cancer is solely contributed by ERMs.

## Methods

### Reagents

Antibodies against protein phosphatase 2 A (PP2A) were purchased from Merck Millipore (Darmstadt, Germany). Antibodies against phospho-PP2A (Tyr^307^), phospho-protein kinase C alpha (PKCα) (Thr^638^), PKCα, phospho-ERM (Thr^567^), and Src were purchased from Abcam (MA, USA). Antibodies against phospho-Src (Tyr^416^) and ERM were purchased from Cell Signaling Technology (Danvers, MA, USA). Antibodies against β-actin were purchased from Sigma-Aldrich (St. Louis, MO, USA). Anti-vimentin antibody and anti-HA tag antibodies were purchased from Santa Cruz Biotechnology (Santa Cruz, CA). Horseradish peroxidase (HRP)-conjugated goat anti-rabbit IgG and goat anti-mouse secondary antibodies were purchased from Enzo Life Sciences (Farmingdale, NY, USA). Resistin was obtained from Phoenix Pharmaceuticals (Belmont, CA). 1,2-Bis(o-aminophenoxy) ethane-N,N,N′,N′-tetraacetic acid (BAPTA)-AM was purchased from Abcam (MA, USA). Gö6976 and PP2 were obtained from Calbiochem (San Diego, CA, USA). Okadaic acid was purchased from Sigma-Aldrich. Protein A-agarose beads were obtained from GE Healthcare (Piscataway, NJ, USA). Fluo-3 AM was obtained from Invitrogen (Leiden, Netherlands). Ezrin-EGFP plasmid was kindly provided to us by Dr. Y. H. Moon (Korea University, Seoul, Korea), and the PKCα-DS-Red plasmid was kindly provided to us by Dr. Kwang-Youl Lee (Chonnam University, Kwangju, Korea).

### Cell culture

Human breast cancer MDA-MB-231 cells (ATCC, Rockville, MD) were cultured in RPMI 1640 medium supplemented with 10% fetal bovine serum (FBS; GIBCO BRL, Carlsbad, CA), 100 U/ml penicillin, and 100 μg/ml streptomycin. Cells were maintained at 37 °C in a humidified incubator.

### Ca^2 + ^measurement

Cells were loaded with 5 μM Fluo-3 AM in regular culture medium for 45 min at room temperature. Cells were then washed and incubated for 15 min in regular media without Fluo-3 AM to complete the de-esterification process. The cells were treated with resistin, and the cultures plates were placed on a temperature-controlled confocal microscope (Zeiss LSM 700 Meta; Zeiss, Oberkochen, Germany) at 10× magnification. The excitation and emission wavelengths for signal detection were 488 nm and 515 nm, respectively.

### Western blot analysis

Following experimental manipulations, the medium was removed, and the cells were washed with ice-cold phosphate-buffered saline (PBS). They were then lysed with 100 μl of lysis buffer [50 mM Tri-HCl (pH 7.4), 1% Triton X-100, 0.25% sodium deoxycholate, 150 mM EDTA, 1 mM sodium orthovanadate (Na_3_VO_4_), 1 mM NaF, 1 mM phenylmethylsulfonyl fluoride (PMSF)]. Proteins were resolved on 10% SDS-PAGE gels and transferred to nitrocellulose membranes. Membranes were blocked in Tris-buffered saline with 0.1% Tween 20 (TBS-T buffer) and 5% dry milk (w/v) for 1 h, and washed 3 times in TBS-T. Membranes were then incubated overnight at 4 °C with primary antibodies, and probed with a secondary antibody conjugated to HRP (Amersham International PLC, Buckinghamshire, UK) for 1 h. The blots were visualized via chemiluminescence using the Amersham Biosciences ECL Detection System (Amersham International PLC).

### siRNA transfection for ezrin knockdown

Transient transfections were performed with Lipofectamine 2000 (Invitrogen, Carlsbad, CA, USA) according to the manufacturers protocol. In brief, 5 μl of siRNA for On-TARGET plus SMART pool ezrin siRNA (L-017370-00-0005) oligonucleotide targeted to human ezrin (GCGCGGAGCUGUCUAGUGA, GCGCAAGGAGGAUGAAGUU, GGAAUCAACUAUUUCGAGA, and GCUCAAAGAUAAUGCUAUG; NM_003379, Dharmacon, Lafayette, CO, USA) and 5 μl of Lipofectamine 2000 were each diluted in 95 μl of reduced serum medium (Opti-MEM; Invitrogen), and then mixed. The mixtures were incubated for 15 min before being added dropwise to a culture well containing 800 μl of Opti-MEM to achieve a final siRNA concentration of 50 nM.

### Wound-healing assay

Confluent cells were serum starved for 12 h, and then a standardized cell-free area was introduced by scraping the monolayer with a sterile tip. Cells were photographed using a phase-contrast microscope (4× , Nikon TS100). After intensive washing, fresh medium supplemented with 1% FBS was added into cells with different concentrations of resistin. After incubation for 48 h, cells were photographed again at 3 random areas. The migrated cells were quantified by manual counting, and the inhibition ratio was expressed as percentage of the control.

### Invasion assay

The upper chamber of a transwell insert (8 μm in pore size) was coated with 100 μl of a 1:6 mixture of Matrigel (BD Biosciences, Bedford, MA) and PBS (invasion assay), and dried for 30 min at 37 °C. Cell suspension (100 μl; 4× 10^5^ cells/ml) in serum-free medium was placed in the upper compartment of the chamber. The bottom chambers were supplemented with 500 μl complete medium (10% FBS) containing the indicated concentrations of resistin. After incubation for 24 h, the non-migrant cells from the upper face were scraped using a cotton swab. The invaded cells on the lower face were fixed with 4% paraformaldehyde (PFA), and stained with crystal violet (Sigma-Aldrich, St. Louis, MO, USA). Random fields were counted, and representative images were photographed using the AxioCam HRC CCD camera (Carl Zeiss).

### Immunoprecipitation

Cellular protein (1 mg) was mixed with 1 μg of anti-PP2Ac (mouse monoclonal antibody) or anti-PKCα (rabbit polyclonal antibody) antibody, and incubated at 4 °C for 24 h. Immune complexes were captured with protein A-Sepharose (Amersham, Uppsala, Sweden) for an additional 3 h. Precipitated immune complexes were washed 3 times with the wash buffer [25 mM HEPES, 5 mM EDTA, 1% Triton X-100, 50 mM NaF, 150 mM NaCl, 10 mM PMSF, 1 μM leupeptin, 1 μM pepstatin, and 1 μM aprotinin (pH 7.2)]. The washed sample was resuspended in SDS sample buffer [125 mM Tris-HCl (pH 6.8), 20% (v/v) glycerol, 4% (w/v) SDS, 100 mM dithiothreitol, and 0.1% (w/v) bromophenol blue] and heated at 100 °C for 5 min.

### Confocal microscopy

MDA-MD-231 cells expressing PKCα-DS-Red or Ezrin-GFP were fixed with 4% PFA/PBS, and permeabilized with 0.2% Triton-X 100. Nuclei were stained with Hoechst 33342 dye for 30 min at 25 °C. Confocal images were obtained using a Zeiss confocal microscope LSM700, and analyzed with the Zeiss LSM image browser software (Carl Zeiss, Oberkochen, Germany).

### Immunofluorescence staining

Cells were fixed with 4% PFA/PBS, and permeabilized with 0.2% Triton-X 100. After blocking with 0.2% bovine serum albumin for 30 min, cells were incubated with anti-PKC-α antibody at 1:500 dilution for 60 min, and probled with Cy3-labeled secondary antibody (Molecular Probe, Eugene, OR, USA). Stained cells were visualized using a Zeiss confocal microscope.

### Data analysis

Statistical analyses were conducted using the Sigma Stat software (SPSS Inc., Chicago, USA). Data are expressed as mean ± SEM. *P* values < 0.05 were considered statistically significant.

## Additional Information

**How to cite this article**: Lee, J. O. *et al.* Resistin, a fat-derived secretory factor, promotes metastasis of MDA-MB-231 human breast cancer cells through ERM activation. *Sci. Rep.* **6**, 18923; doi: 10.1038/srep18923 (2016).

## Supplementary Material

Supplementary Information
