# Supplementary material for: Resistin, a fat-derived secretory factor, promotes metastasis of MDA-MB-231 human breast cancer cells through ERM activation
Source: Sci Rep. 2016 Jan 5;6:18923. doi: 10.1038/srep18923 (PMC4700449; doi:10.1038/srep18923)

Resistin, a fat-derived secretory factor, promotes metastasis of MDA-MB-231 human breast cancer cells through ERM activation

Jung Ok Lee, Nami Kim, Hye Jeong Lee, Yong Woo Lee, Su Jin Kim, Sun Hwa Park, Hyeon Soo Kim*

*Department of Anatomy, Korea University College of Medicine, Seoul, Korea*

**Running title:** Resistin promotes metastasis via ERM activation

***Address correspondence to:** Hyeon Soo Kim, MD, PhD, 126-1, Anam-dong 5-ga, Seongbuk-gu, Seoul, Korea. Tel: 82-2-2286-1151; Fax: 82-2-920-5696; E-mail: anatomykim@korea.ac.kr

**Supplementary Fig. 1A. Resistin increases the phosphorylation of Src in MCF-7 breast cancer cells.**

MCF-7 cells were treated with different doses of resistin and cultured for 1 h. The cell lysates (30 μg) were analyzed by western blotting using an antibody against phospho-Src, while Src served as a control.

**Supplementary Fig. 1B. Resistin increases the phosphorylation of PP2A in MCF-7 breast cancer cells.**

MCF-7 cells were treated with different doses of resistin and cultured for 1 h. The cell lysates (30 μg) were analyzed by western blotting using an antibody against phospho-PP2A, while PP2A served as a control.

**Supplementary Fig. 1C. Resistin increases the phosphorylation of PKCα in MCF-7 human breast cancer cells.**

MCF-7 cells were treated with different doses of resistin and cultured for 1 h. The cell lysates (30 μg) were analyzed by western blotting using an antibody against phospho-PKCα, while PKCα served as a control.

**Supplementary Fig. 1D. Resistin increases the phosphorylation of ERM in MCF-7 breast cancer cells.**

MCF-7 cells were treated with different doses of resistin and cultured for 1 h. The cell lysates (30 μg) were analyzed by western blotting using an antibody against phospho-ERM, while ERM served as a control.

**Supplementary Fig.1A .** Resistin increases the phosphorylation of Src in MCF-7 breast cancer cells.

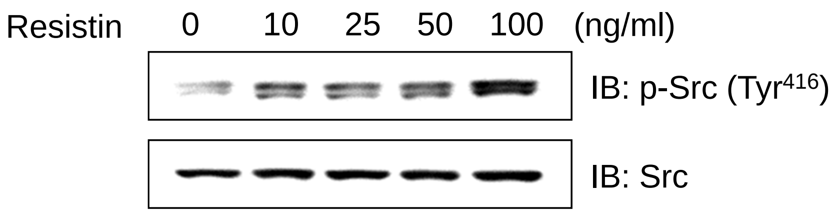


**Supplementary Fig.1B .** Resistin increases the phosphorylation of PP2A in MCF-7 breast cancer cells.

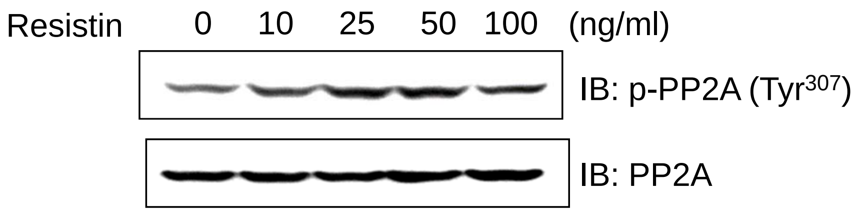


**Supplementary Fig.1C .** Resistin increases the phosphorylation of PKCα in MCF-7 breast cancer cells.

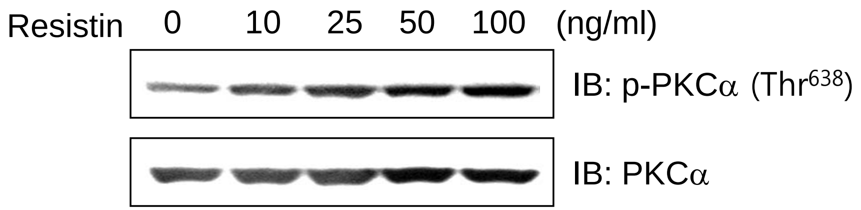


**Supplementary Fig.1D .** Resistin increases the phosphorylation of ERM in MCF-7 breast cancer cells.

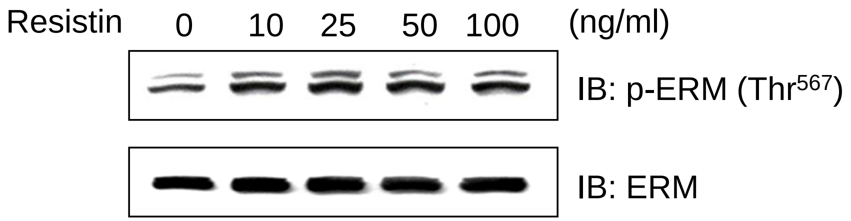

Supplement: Supplementary Information [file srep18923-s1.pdf]
